# A LC–MS method for 25-hydroxy-vitamin D3 measurements from dried blood spots for an epidemiological survey in India

**DOI:** 10.1038/s41598-020-76955-w

**Published:** 2020-11-16

**Authors:** Rashmi Lote-Oke, Jwala Pawar, Shriram Kulkarni, Prasanna Sanas, Neha Kajale, Ketan Gondhalekar, Vaman Khadilkar, Siddhesh Kamat, Anuradha Khadilkar

**Affiliations:** 1 Hirabai Cowasji Jehangir Medical Research Institute, Pune, India; 2 Department of Biology, Indian Institute of Science Education and Research, Pune, India

**Keywords:** Biochemistry, Molecular biology, Endocrinology

## Abstract

Vitamin D, a secosteroid, plays an important role in several physiological processes, and its deficiency can lead to numerous pathophysiological conditions in humans. The primary objective of this study was to develop and validate the robustness of a mass spectrometry-based method capable of quantifying 25(OH)D3 for an upcoming epidemiological survey in India and to pilot test it on healthy volunteers. We first describe the development and validation of various experimental parameters that ascertain the robustness and reliability of 25-hydroxy-vitamin D3 (25(OH)D3) extractions and quantitative measurements from Dried Blood Spot (DBS) samples, where we used eight disks of 3 mm each, punched from the circular spot covering the entire circumference of the spot. Next, we conducted a pilot study, comparing 25(OH)D3 levels from serum and DBS samples from 45 participants using a protocol developed for specifically this purpose. We found that the mean 25(OH)D3 concentrations in DBS samples were comparable to the serum levels (P > 0.05). In summary, our extraction and LC–MS protocol for quantitative 25(OH)D3 measurements are robust and reproducible, and will serve as an invaluable tool for upcoming epidemiological surveys in India and perhaps around the world.

## Introduction

Vitamin D is a secosteroid that plays a significant role in bone mineralization and remodelling by regulating the calcium and phosphorus homeostatic balance^1^. Recent reports suggest that it also plays an important role in several other vital physiological functions such as the protection against respiratory tract infections, muscle contractions and nerve conduction^2^. Humans typically obtain vitamin D from two sources: exposure to sunlight which converts 7-dehydrocholesterol (7DHC) present in epidermal layer of skin into cholecalciferol (vitamin D3) and consumption of vitamin D rich foods such as certain fatty fish, shitake mushrooms and yeast which provide ergocalciferol (vitamin D2)^3^. In humans, both ergocalciferol and cholecalciferol are hydroxylated by hepatic hydroxylases to 25 hydroxy-vitamin D [25(OH)D2 and 25(OH)D3)]^4^. Being the primary storage form with longer half-life, the concentration of 25-hydroxy-vitamin D is measured to determine the physiological vitamin D status^5^.

Vitamin D bioavailability from commonly consumed foods is very low in most populations, particularly when the foods are not fortified. Therefore, the primary source for acquiring vitamin D in most populations is through sunlight exposure, and hence understandably, populations with limited sunlight availability (especially in winter seasons) are more prone to its deficiency. Interestingly, vitamin D deficiency has also been reported in countries located in lower latitudes, where there is no scarcity of sunlight, with India being one of these nations. The majority of the Indian population lives in the region of ample sunshine throughout the year (8.4∘ to 37.6∘ north latitude), and despite this, studies have reported vitamin D deficiency in different age groups and geographical locations of India^4^.

Despite the lack of consensus within the scientific community on desired physiological concentrations of vitamin D, the most commonly accepted definition for vitamin D deficiency indicates a blood concentration less than 20 ng/mL (50 nmol/l) in adults^6^. While there are several studies that have consistently reported high prevalence of vitamin D deficiency globally, comparable population scale studies in an Indian population, are to the best of our knowledge, not performed, and hence unavailable.

Thus, the primary purpose of the current study was to develop an efficient and robust epidemiological protocol for assessment of vitamin D in various representative groups of the Indian population using dried blood spots (DBS). Dried blood spotting is minimally invasive and a resource friendly alternative to serum/plasma samples, especially in large epidemiological studies. Being minimally invasive it is also the method of choice for paediatric and geriatric populations^7^. The specific objectives our study were (1) to develop and validate the robustness of a mass spectrometry-based method capable of quantifying 25(OH)D3 that we intend using for subsequent epidemiological studies, (2) to assess 25(OH)D3 concentrations in serum and DBS (using the newly developed method) in a pilot sample of healthy adult volunteers.

## Materials and methods

### Chemicals

Analytical standards for 25(OH)D3 were purchased from Sigma-Aldrich. LC–MS grade acetonitrile, methanol, and hexane were purchased from J.T. Baker (Thermo Fisher Scientific). Double deionized water was purchased from HiMedia Laboratories. Stock solutions of 25(OH)D3, and [^2^H_6_]-25(OH)D3 were prepared at a concentration of 1 mg/mL in ethanol and stored at − 80 °C until use.

### Blood sampling and spotting

Healthy adult volunteers (age: 22 to 48 years) were requested to provide blood samples for the study, as per a protocol which was approved by the Institutional Ethics Committee- Jehangir Clinical Development Centre Private Limited. We confirm that all the experiments in this study are in accordance with Declaration of Helsinki, Institutional Ethics committee and Institutional scientific committee. All volunteers provided an informed consent to participate in the study. Volunteers were examined by a physician on site to confirm absence of any disease before sampling was performed. Samples were collected according to the guidelines in the Declaration of Helsinki for preparation of vitamin D free artificial blood and for assessment of vitamin D from serum and DBS.

### Preparation of vitamin D free artificial blood

Blood samples (3 mL each) were collected in plain and EDTA tubes from 25 healthy volunteers in a community health camp, pooled and centrifuged at 2500 rpm for 10 min at room temperature to separate the samples into serum/plasma and cellular components. The serum was aliquoted into separate tubes and conserved for preparation of vitamin D free serum. Cellular components (red blood cells, leukocytes and thrombocytes) were washed with 25 mL phosphate-buffered saline (PBS) solution (pH 7.4), followed by centrifugation at 2500 rpm. This step was repeated 5 times. The pooled serum was then mixed thoroughly with activated charcoal (0.14 gm/10 mL of serum) by shaking at 280 rpm for 8 h. The mixture was centrifuged at 10,000 rpm for 25 min. The supernatant was separated, the centrifugation step was repeated, and the remaining serum was collected by filtration. The filtered charcoal treated serum (2 mL) was stored at − 20 °C until use^8^. The PBS treated cellular components and the charcoal treated serum were mixed together in a ratio of 37:63 (v/v) based on the natural composition of female whole blood (using mean adult female haematocrit values from a previous study) resulting in 10 mL of artificial vitamin D-free whole blood as a homogenous mixture of serum and blood cells^5^.

### Blood spot (DBS) sample preparation

From each of the 45 study participants, intravenous samples were collected (approximately 3–4 mL) using plain and Heparin tubes. Immediately after collection, 3 mL whole blood from both tubes was aliquoted in an appropriate tube and Calcifediol-d_6_ (15 µL) was added to it to achieve a concentration of 5 ng/mL, ensuring thorough mixing. DBS cards were prepared by pipetting 55 µL of whole blood with [^2^H_6_]-25(OH)D3 on each spot. The blood spots were then allowed to dry at room temperature for 2 h and were subsequently transferred to light protective storage polythene bags along with a desiccant. The bags were stored at − 20 °C until extraction. 500 µL of whole blood was separately aliquoted and used for Haematocrit analysis using Yumizen H 500 (Horiba, India). Approximately 30 min after the addition of [^2^H_6_]-25(OH)D3, the plain and heparin tubes were centrifuged for 10 min at 2500 rpm and aliquots of 500 µL of serum/plasma were prepared. All the aliquots were flash frozen in liquid nitrogen and stored at − 80 °C freezer until extraction. Samples were neither subjected to prolonged light exposure nor multiple freeze/thaw cycles.

### Matrix matched calibration

#### For serum

The charcoal treated serum was used for matrix calibration. Each tube containing 0.5 mL charcoal treated serum was spiked with 25(OH)D3 spanning a concentration range of 2.25, 11.25, 22.5, 67.5, 112.5, 225 ng/mL.

#### For DBS

The blank DBS cards (Whatman filter paper number 903) were prepared by spotting 55 µL of artificial blood in each spot. Eight discs of 3 mm diameter were punched into a 2 mL Eppendorf tube from the DBS. Each tube was spiked with 10 µL of 25(OH)D3 spanning a concentration range of 6.4, 9.6, 25.6, 51.2, 102.4,153.6 and 204.8 ng/mL.

### Extraction of vitamin D

#### For serum

The serum extraction method was a modification of a previously described method^9^. To the 500 µL serum sample a mixture of 200 µL of Acetonitrile: Methanol (70:30) was added. The tube was briefly vortexed and then sonicated for 30 min (Labman Scientic Instruments, Chennai, India). After sonication, 1 mL hexane was added followed by brief vortexing and centrifugation at 10,000 rpm for 10 min. 900 µL of hexane was carefully transferred to a fresh tube without disturbing the phase separation. The hexane was evaporated to dryness using nitrogen concentrator (KeMi scientific, Pune India). The residue was reconstituted in 200 µL of methanol followed by vortexing to ensure uniform mixing.

#### For DBS

DBS extraction method was a modification of a previously described method^10^. A circular disc of 3 mm diameter (corresponding to 3.2 µL of whole blood) was punched from a spot on the DBS card using a semi-automated puncher (Horizon Speciality, USA). For the assay, 8 discs from each participant were used (corresponding to 25.6 µL of whole blood). The punch was cleaned with 70% ethanol prior to and after taking sample from each study participant. The discs were placed into 2 mL Eppendorf tubes along with 250 µL of water. Each tube was vortexed for 30 s and then sonicated for 1 h using a sonicator (Labman Scientific Instruments, Chennai, India). 250 µL of methanol was added to each tube and the tubes were sonicated for another 30 min. Subsequently, 500 µL of hexane was added to each tube followed by centrifugation at 10,000 rpm for 10 min. Approximately 400 µL of hexane was carefully aspirated in a fresh tube without disturbing the phase separation and evaporated to dryness using nitrogen concentrator (Kemi Scientific, Pune India). The residue was reconstituted in 25 µL of methanol followed by vortexing to ensure uniform mixing.

#### LC–MS/MS

The LC–MS system used was Shimadzu 8045 triple quadrupole mass spectrometer (MS) with the electrospray ionization (ESI) source in positive ion polarity mode fitted with Nexera X2 LC-30A UHPLC system. Data were collected in the multiple reaction monitoring (MRM) mode monitored for various vitamin D components in the extract using the following MS parameters: oven temperature 30 ∘C, interface temperature 298 ∘C, desolvation line temperature 196 ∘C, nebulizing gas flow 3.0 L/min, heating gas flow 10.0L/min, drying gas flow 18.0 L/min, IG vacuum 1.7e − 003 Pa, PG vacuum 6.9e + 001 Pa, CID Gas 230 KPa. Each vitamin D3 species and internal standard was identified and quantified using three MRM transitions reported previously, that for the various species were: (i) 25(OH)D3 = 401.2 → 383.3, 401.2 → 365.4, 401.2 → 159.1,; (ii) vitamin D3 = 385.2 → 259.2, 385.2 → 241.0, 385.2 → 107.3; and (iii) the internal standard [^2^H_6_]-25(OH)D3 = 407.5 → 389.5, 407.5 → 371.0, 407.5 → 107.0^9^. Isolation and separation of analytes by chromatography was achieved by using a Shimadzu –Premier C18 150 mm × 2.1 × 3 µm column without derivatization. A typical LC–MS consisted of 10 min, with mobile phase A = water + 0.1% formic acid, and B = methanol + 0.1% formic acid, with a flow rate of 400 μL/min, and had the following gradient sequence: (i) equilibration with solvent B at 90% from 0.0 to 1.4 min, (ii) linear increase in solvent B from 90 to 100% from 1.5 to 4 min, and (iii) equilibration and washing with solvent B at 90% from 4.1 to 10 min. MS acquisition was stopped after 5 min of the LC gradient, and the remaining eluent was directed to waste collection system.

### Statistical analysis

SPSS software was used for data analysis (version 26 0.0, IBM statistics data editor, IBM Corp., released 2017. Armonk, NY). Variables to be tested were assessed for normality using Kolmogorov–Smirnov KS test. Depending on the normality, appropriate tests e.g. independent sample t-test for normal and Wilcoxon Signed Rank Test (for non-normal variables) were applied. For categorical variables, Chi square test was used for reporting associations between two categorical variables. Bland Altman plot was constructed to assess differences between the 25(OH)D3 concentrations assessed using the two methods, i.e. from serum and DBS. Level of significance was set at P < 0.05.

### Ethics declaration

Ethical Approval was obtained from the Institutional ethics committee (Jehangir Clinical Development Centre Private Limited, Approval dated 21/06/2016). We confirm that all the experiments in this study are in accordance with Declaration of Helsinki, Institutional Ethics committee and scientific committee. All volunteers provided an informed consent to participate in the study.

## Results

### Validation of LC–MS/MS method

**Figure 1:**
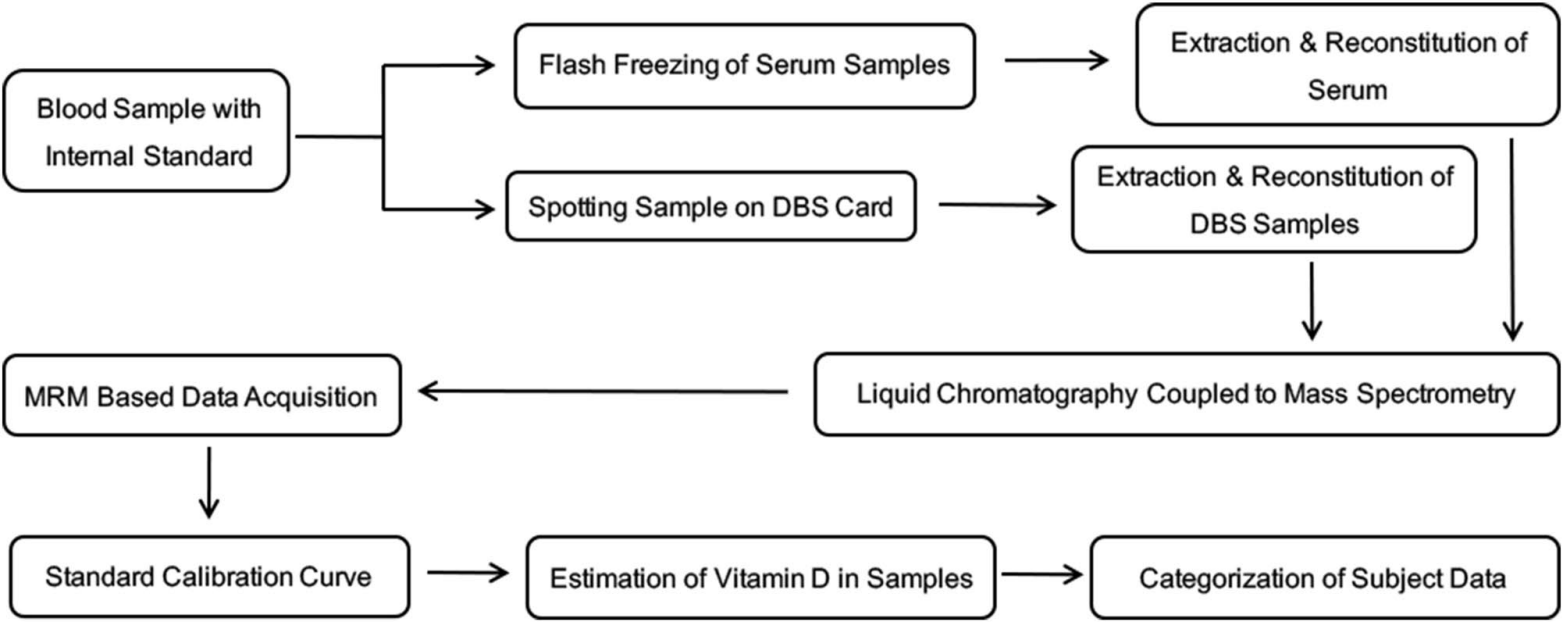
Sample analysis workflow. Blood collection in study participants, the serum was separated from blood and flash freezing was carried out in liquid nitrogen, uncoagulated blood was spread on DBS card and allowed to dry completely at room temperature. Both samples were extracted on the same day using respective extraction protocols followed by LCMS analysis in Shimadzu 8045. Matrix matched calibration was performed using charcoal treated serum for serum samples and vitamin D free artificial blood for DBS samples and standard calibration curves were generated for both. The circulating vitamin D levels were estimated based on these standard calibration curves.

The LC–MS/MS method described here was validated based on the procedures described in the ‘Bioanalytical method validation Guidance for Industry’ of the U S Food and Drug Administration^11^. A schematic workflow of the steps involved in the method validation is presented in Fig. 1. Since this assay was adapted in part from a previously reported and validated method^10^, only a partial validation of this method using the fit for purpose concept was performed here.

**Figure 2:**
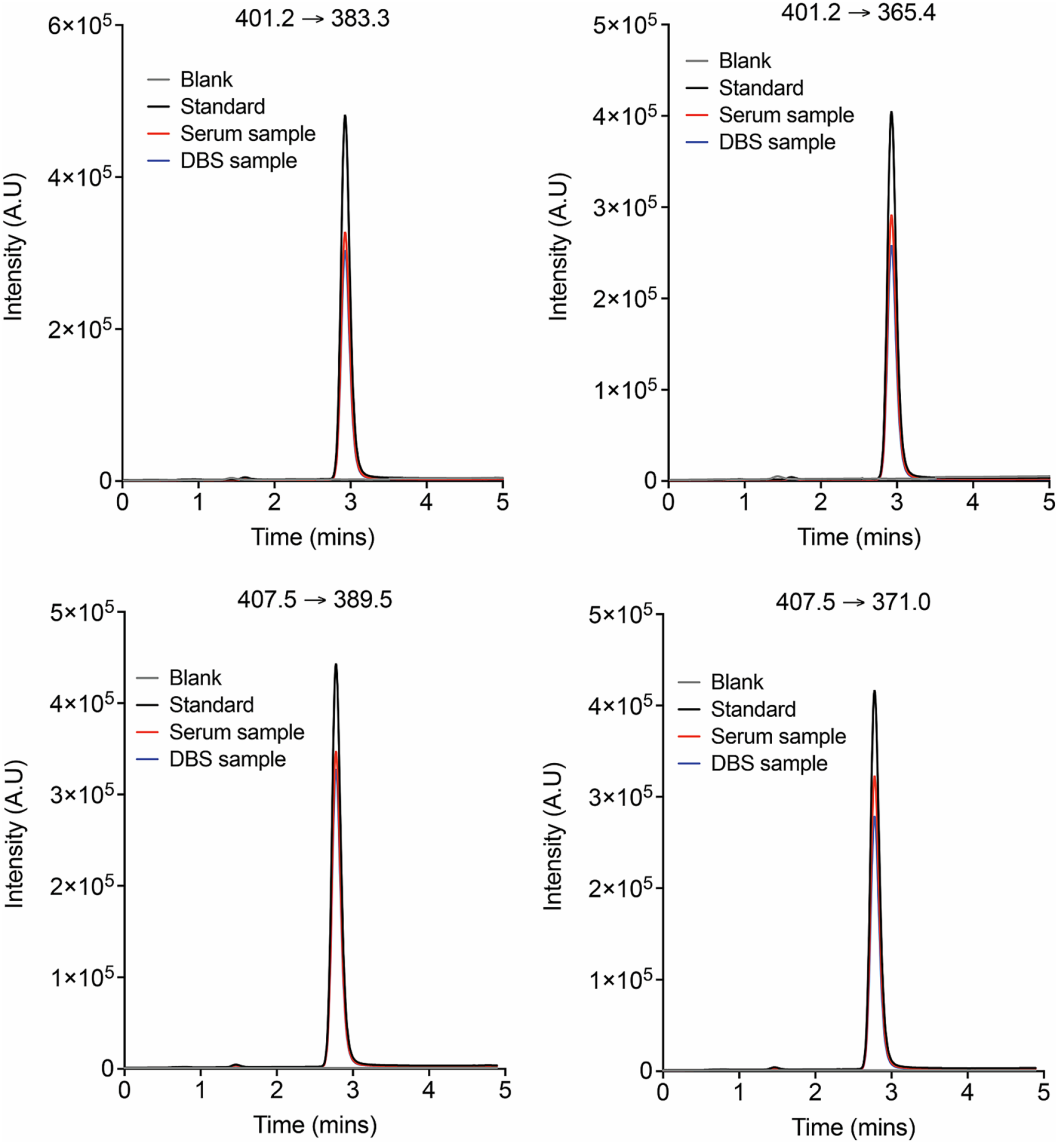
Representative spectra for the qualifier and quantifier MRM of 25(OH)D3 (analyte; m/z = 401.2), and [^2^H_6_]-25(OH)D3 (internal standard; m/z = 407.5).

**Figure 3:**
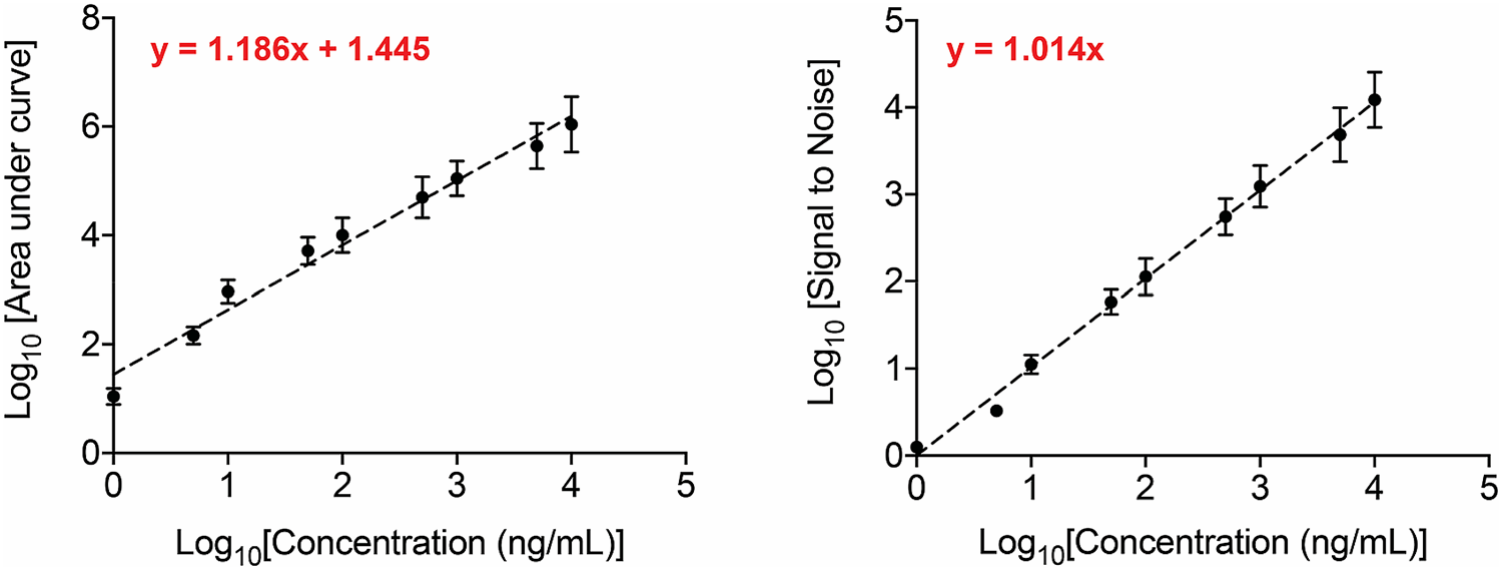
Plots displaying linearity, dynamic range, and signal to noise ratio for 25(OH)D3 measurements using the LC–MS method.

The method was selectivity tested by comparing chromatograms from five independent samples of vitamin D free serum and artificial DBS with and without spiking [^2^H_6_]-25(OH)D3 or 25(OH)D3. As the samples contained endogenous 25(OH)D3, area ratios of qualifier MRM transition 401.2 → 383.3 to quantifier MRM transition 401.2 → 365.4 were calculated and used for the assessment of selectivity for the detection of 25(OH)D3. Representative spectra for the qualifier and quantifier MRM for both the analyte 25(OH)D3, and the internal standard [^2^H_6_]-25(OH)D3 are presented in Fig. 2. Carry over was assessed by measuring blank samples after analysis of every high-content sample, and no significant carryover (< 1%) was observed in our method. Linearity and dynamic range of this LC–MS/MS method were also determined by analysis of calibration samples (Fig. 3), and were found to pass set experimental thresholds (Table 1). The limit of quantification (LOQ) and the limit of detection (LOD) for 25(OH)D3 were set to a signal to noise ratio threshold of 10 and 5 respectively, and were found to be approximately 10 and 5 ng/mL respectively (Fig. 3). Differences between nominal concentrations and calculated concentrations were determined and expressed in percentage. Deviations of the measured values from nominal values should be ≤ 20% for the LOQ (LOQ) and ≤ 15% for the remaining calibration points. At least 75% of the calibration levels on each day need to meet these criteria. As calibration was performed with endogenous samples, the following concentration range was tested: 2·25–225 ng/mL calculated as serum concentrations. Details of the parameters assessed, along with their acceptance criteria, and the experimental outcome for this method for quantification of 25(OH)D3 can be found in Table 1.

**Table 1:** Description of the parameters for validation of the LC–MS/MS method for quantification of 25(OH)D3.

Validation element	Description	Acceptance criteria	Results
Selectivity	Minimum interference in the detection of analyte of interest ensured by assessing matrix effect on ion suppression, ion enhancement or extraction efficiency	Blank and zero calibrators should be free of interference at the retention times of the analyte and IS Spiked samples should be ± 20% LOQ The IS response in blank should not exceed 5% of the average IS response of the calibrators and QCs	Pass
Carry-over	Response of analyte due to the previous injection	Carry over should not exceed 20% of LOQ	Pass
Accuracy and precision	To ensure the extraction protocol is efficient and reproducible	± 20% of nominal concentration at LOQ and ± 15% for the others %RSD ± 20% at LOQ and other levels	< ± 20% RSD at LOQ and < ± 15% RSD at other concentrations
Linearity & dynamic range	Concentration range accepted in the particular study	Non zero calibration should be ± 15% of nominal concentrations and for LOQ calibrators should be ± 20% 75% and a minimum of six non zero calibrator levels should meet the above criteria in each calibration run 3-orders of linear range	Pass

*LOQ* limit of quantification, *RSD* residual standard deviation.

### Assessing the matrix effect and inter-day variance of the method

Although accepted as the gold standard, LC–MS/MS analysis of 25(OH)D3 from serum is not a universal assay and variability in sample preparation, chromatographic separation and ionization/fragmentation have been reported^12^. Matrix effects (ion suppression/ion enhancement) are recognized as one of the major potential sources of errors in serum LC–MS/MS analysis of 25(OH)D3, and hence it has been recommended that the matrix effects for any LC–MS/MS measurement of 25(OH)D3 should be evaluated thoroughly^12^. Also, the ionization efficiency of 25(OH)D3 is known to be low and in the absence of derivatization relatively larger volume of serum/plasma (0.1–0.5 mL) need to be used. Similarly, for DBS samples it has been reported that the composition of blood and its volume dramatically affects metabolite measurements from DSB samples. Hence, to overcome any matrix effects, matrix matched calibration using artificially prepared 25(OH)D3 free blood is recommended^5^.

For the current study, we performed matrix matched calibration using charcoal treated serum for serum samples and artificially prepared blood using same charcoal treated serum for DBS samples. Considering the fact that 25(OH)D3 is hydrophobic in nature and is tightly bound to vitamin D binding protein(s), its extraction efficiency from serum may be affected. To ensure the maximum extraction efficiency in serum samples, we used a two-pronged strategy. Firstly, after separation of serum, the samples were flash frozen using liquid nitrogen. The goal of flash freezing was to minimize any potential biochemical reactions in serum that may result in metabolism/degradation of serum 25(OH)D3. Secondly, in a subset of the experimental samples (n = 31), a known concentration of internal standard ([^2^H_6_]-25(OH)D3) was spiked in to establish an extraction recovery factor. Addition of internal standard was followed by separation as serum. The peak areas of the internal standard ([^2^H_6_]-25(OH)D3) were obtained at the end of extraction and were used to calculate the % recovery of [^2^H_6_]-25(OH)D3 in each sample. To ensure a uniform recovery calculation, same procedure was used to calculate recovery from corresponding DBS samples. The mean recovery for serum sample was found to be 79.03% while that for the corresponding DBS sample was found to be 69.72%. The % mean recovery was used to calculate the recovery factor (100/mean % recovery), and these were set at 1.27 and 1.43 for serum and DBS samples respectively.

For DBS samples, the measured 25(OH)D3 concentration needed to be normalized to its % haematocrit. The % haematocrit for the artificial blood used for calibration was 37%, and the following formula (Eq. 1) was used for normalizing the 25(OH)D3 concentrations for DBS samples that were used in these calibration experiments. For experimental (subject) DBS samples, the 25(OH)D3 concentration was further corrected for individual haematocrit (HCT) value for each participant by applying the respective correction factor (Eq. 2). Hence, for the experimental (subject) DBS samples, the final 25(OH)D3 concentration was eventually obtained by multiplying Eq. (1) by the individual HCT correction factor that was obtained from Eq. (2).

1 $$ \left[ {{25}\left( {{\text{OH}}} \right){\text{D3}}} \right]_{{{\text{normalised}}}} = \, \left[ {{25}\left( {{\text{OH}}} \right){\text{D3}}} \right]_{{{\text{measured}}}} \times \, \left( {{1}00 - {37}} \right)/{1}00 $$

2 $$ {\text{Individual HCT Correction Factor }} = \, \left( {{1}00 - {37}} \right)/ \, \left( {{1}00 - \% {\text{HCT for participant}}} \right) $$

**Figure 4:**
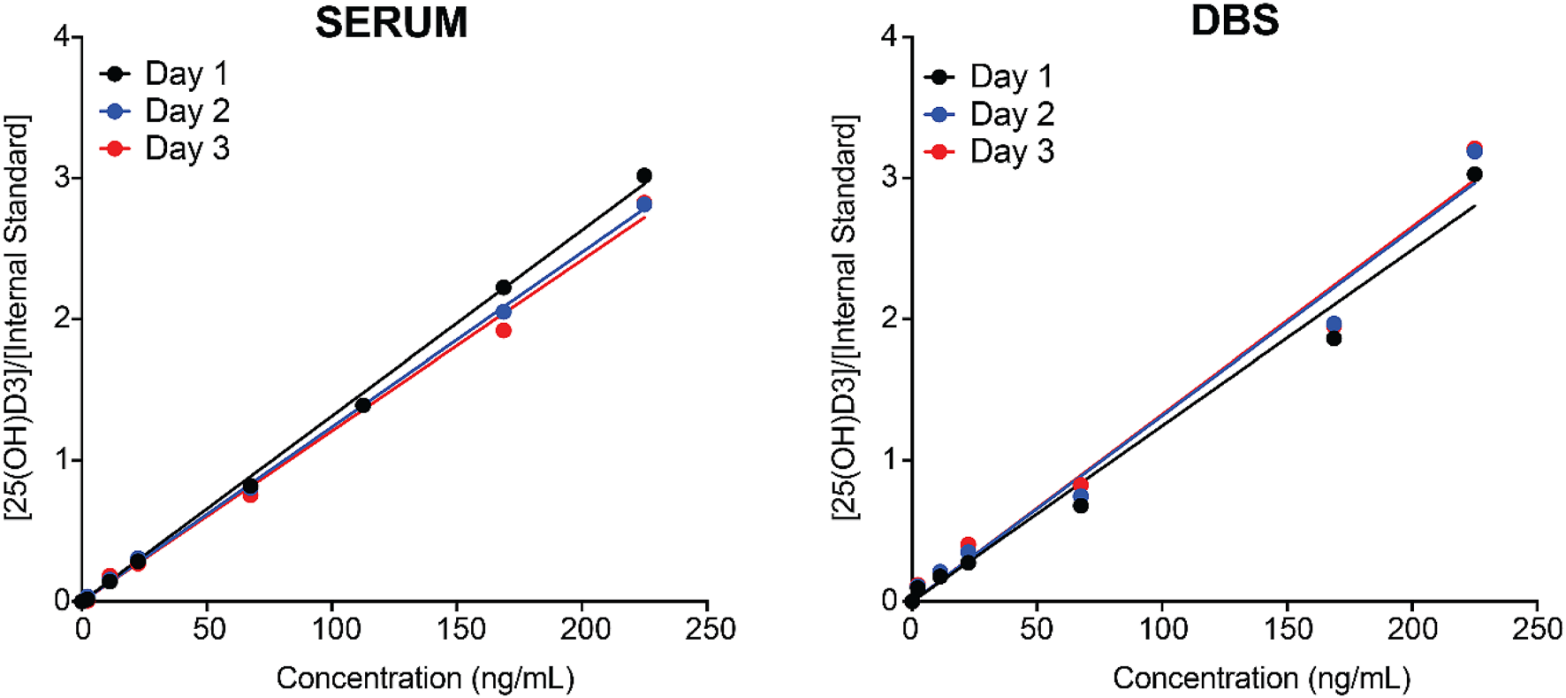
Inter-day accuracy and precision for serum and DBS samples.

Next, for assessing accuracy and inter-day variance of this method, three samples representing low, medium and high concentrations with five aliquots of each were analysed on 3 different days for both serum and DBS samples. The artificial blood with 37% haematocrit using charcoal treated serum was used as a blank for both serum and DBS samples. To estimate inter-day precision, triplicates of each sample were analysed on three different days, and these were found to be identical in this method (Fig. 4).

### Human subject data analysis

**Figure 5:**
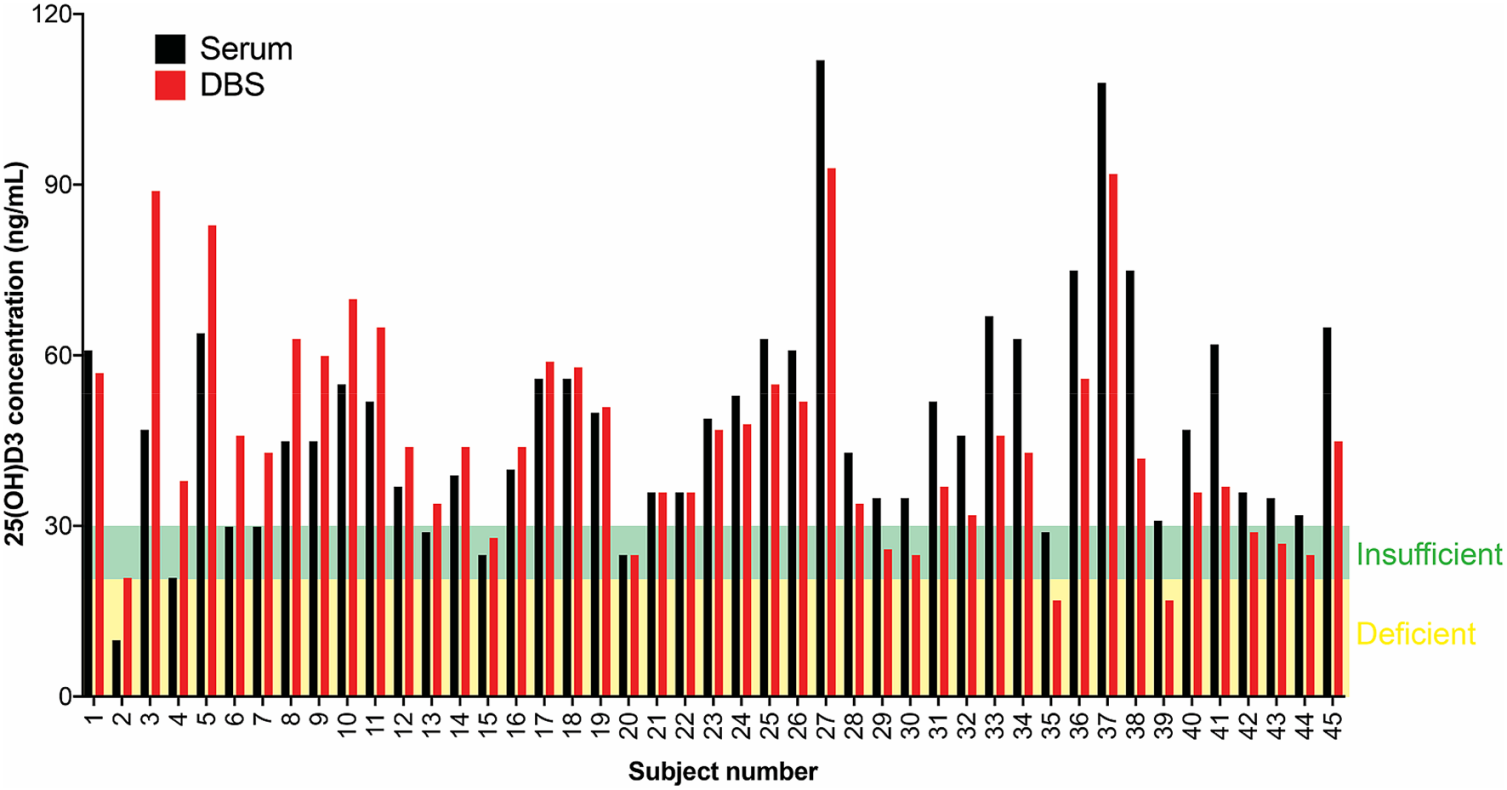
Pilot Study to analyse circulating vitamin D levels using DBS samples.

Finally, to validate the robustness of our LC–MS/MS method in measuring 25(OH)D3 from human samples, we decided to perform a small pilot study in healthy human volunteers. In this pilot study, serum and DBS samples were extracted and analysed from 45 healthy volunteers, with a mean age ~ 37 years, mean body mass index = 24.5 kg/m^2^ (males = 23). Mean vitamin 25(OH)D3 concentrations in serum and DBS samples from these human subjects were 47 ± 19 and 44 ± 19 ng/mL respectively. As the primary aim of our study was to develop an efficient epidemiological protocol for assessment of 25(OH)D3 concentrations in a representative Indian population, we classified the samples as vitamin D deficient (vitamin 25(OH)D3 levels < 20 ng/mL), insufficient (vitamin 25(OH)D3 < 30 ng/mL) and vitamin D sufficient (vitamin 25(OH)D3 > 30 ng/mL) by both the serum and DBS methods (Fig. 5). We found that there were no significant differences in the proportion of subjects classified as deficient, insufficient and sufficient by using either serum or DBS samples for the assessment of 25(OH)D3 (Fig. 5) (P > 0.1). Further, we observed that by using this method to assess 25(OH)D3 concentrations in serum or DBS samples, the Pearson Chi square values were similar to each other (Ψ^2^ = 8.63, df = 4, P = 0.07), thus validating the robustness of our method. In accordance with a previously reported analysis for such data, we also assessed these findings statistically using a Bland–Altman plot, and found the serum and DBS data to be in good accordance, and within experimental error limits^9^.

## Discussion

In humans approximately 95% of vitamin D3 is synthesized in epidermal keratinocytes from 7DHC^13^. The B-ring of 7DHC absorbs UVB radiation in the range of 280–320 nm which breaks the bond between C9 and C10 resulting in formation of pre-vitamin D3. Thermal isomerization converts pre-vitamin D3 to D3^14^. The vitamin D3 is hydroxylated at 25th position by hepatic CYP27A1 or CYP2R1 to produce 25 (OH)D3). The 25(OH)D3 is subsequently hydroxylated at C1α in kidneys and other peripheral tissues by CYP27B1 to form 1,25(OH)_2_D3 which is the biologically active form of vitamin D^14^. Another cytochrome P450 enzyme (CYP11A1) catalyses sequential hydroxylation of D3 side chain to form several vitamin D metabolites such as 20(OH)D3, 22(OH)D3, [2O,22(OH)2D3], [20,23(OH)2D3] and [17,20,23(OH)D3]^15^. Among these, 20(OH)D3 is the predominant metabolite of this pathway, that can be detected in serum at an approximately 20 times lower concentration compared to D3^13^.

Prolonged exposure to UVB can cause photo-isomerization of pre-vitamin D3 to tachysterol3 (T3) which undergo UVB driven conversion to sterol3 (L3). Formation of T3 and L3 prevents excessive formation of pre-vitamin D3 and thus protects from D3 intoxication. Recently it has been reported that L3 undergoes CYP11A1 mediated hydroxylation and the hydroxylated lumisterol derivatives provide protection against UVB damage. The hydroxylumisterol derivatives are reported to be present in serum suggesting additional biological activity^16,17^. The CYP11A1 derivatives can be further hydroxylated at C1α by CYP27A1, CYP24A1, CYP11A1, CYP2R1 and/or CYP2A4. It has also been reported that CYP11A1 derived 20,23(OH)_2_D3 and 1,25(OH)_2_D3 induce some overlapping gene expression patterns in keratinocytes^18^. CYP11A1 derived compounds i.e. both the D3 and L3 derivatives display anti-proliferative, anti-inflammatory, anti-cancer and pro-differentiation properties^17^.

Physiologically and clinically, vitamin D status refers to the concentration of hydroxylated D3 and D2 together, and the most widely accepted biochemical marker for assessing systemic vitamin D status is the total serum 25-hydroxyvitamin D [25(OH)D] concentration^19^. For the past decade, quantitative vitamin D analysis from serum/plasma using LC–MS has been considered the gold standard given its sensitivity, precision and lowered susceptibility to matrix effect compared to cell-based assays^10^. Previously published literature has reported that measurement of 25(OH)D3 from DBS using LC–MS/MS is a valid alternative to conventional methods of quantifying vitamin D metabolites from plasma/serum samples. However, being a relatively new and less explored method of analysis, vitamin D analysis from DBS samples shows considerable variability and optimization studies regarding this method are lacking in literature^20^. The primary goal of our study was to optimize and standardize a method for analysis of 25(OH)D3 using DBS for an upcoming epidemiological study in representative Indian population. For any clinical (or epidemiological) assay, the most important performance parameters to be considered are sensitivity, specificity, throughput and labour for sample preparation^21^. Derivatization i.e. modification of original chemical structure of analyte with diverse tags^22^ is commonly used to improve the sensitivity of the vitamin D; however, it makes the assay laborious and may hamper accuracy by forming multiple stereoisomers^10^. Hence, here, we focused on developing an efficient, high throughput method for screening vitamin D deficiency without using chemical derivatization.

It has been estimated that in the Indian population, sunlight exposure leading to indigenous synthesis of D3 is the primary determinant of vitamin D status as it contributes to > 90% of the total 25(OH)D concentration^23^. It has also been reported that, in a population that consumes food items not being fortified with vitamin D, 25(OH)D2 levels were below the detectable limit and hence its assessment of agreement between DBS and serum samples cannot be implemented in an Indian population^24^. Therefore here, we decided to assess the relative concentration of only 25(OH)D3 in DBS and serum, and validate a method for the same. There is a common agreement in the scientific community that 25(OH)D3 is a difficult analyte to quantitate and thus methods for the same are sparse in literature^25^. As a result, the implementation of novel LC–MS/MS based methods in a clinical lab is challenging due to the lack of agreement in serum and DBS samples. However, for an epidemiological study, with the primary aim of reporting prevalence of vitamin D deficiency, the goal should be to accurately report the vitamin D deficiency/sufficiency status compared to a diagnostic protocol where the aim is to derive the method for analysis of vitamin D in DBS that can closely reflect the physiological vitamin D status as closely possible to serum/plasma samples. The LC–MS/MS we report here, does all the above, and fits the bill for us to implement this for our upcoming epidemiological survey.

Despite its robustness, our method still has a few limitations. Here, we have used a C18 stationary phase and this cannot separate isomeric and isobaric vitamin D metabolites. For example, a C18 column can achieve only partial separation of 25(OH)D3 and 25(OH)D2 as well as between 25(OH)D3 and its C3-epimer^7^. As a result, our method may sometimes lead to the overestimation of 25(OH)D concentrations. The accuracy of estimation of vitamin D requires selection of columns that offer high chromatographic selectivity prior to MS identification. However, this strategy sacrifices high throughput due to additional time requirement. Pentafluorophenyl (PFP) column which requires comparatively shorter run time can perhaps be used^7^ but will be substantially more expensive, additionally, it may not be economically feasible for a population scale study. Lastly, a 2-step derivatization i.e. a Diels–Alder reaction with 4-phenyl-1,2,4-triazoline-3,5-dione (PTAD) followed by acetylation, may be used^7^. However, derivatization can compromise accuracy because of its own limitations as discussed in the previous section. Selection of C18 column was a decision in interest of high throughput and cost-effective method. We acknowledge that because of these limitations, the protocol may be used for a population based epidemiological study rather than for diagnostic purposes.

## Data Availability

The datasets generated during and/or analysed during the current study are available from the corresponding author on reasonable request.
