# Author Correction: A LC–MS method for 25-hydroxy-vitamin D3 measurements from dried blood spots for an epidemiological survey in India

**DOI:** 10.1038/s41598-021-87275-y

**Published:** 2021-04-06

**Authors:** Rashmi Lote-Oke, Jwala Pawar, Shriram Kulkarni, Prasanna Sanas, Neha Kajale, Ketan Gondhalekar, Vaman Khadilkar, Siddhesh Kamat, Anuradha Khadilkar

**Affiliations:** 1 Hirabai Cowasji Jehangir Medical Research Institute, Pune, India; 2 Department of Biology, Indian Institute of Science Education and Research, Pune, India

Correction to: *Scientific Reports* 10.1038/s41598-020-76955-w, published online 16 November 2020

This Article contained an error, where the scheme titled “Sample analysis workflow” in the original Figure 1 was adapted without permission from a figure credited to Dr. V. Panchagnula (NCL, Pune). We thank Dr. V. Panchagnula for bringing this inadvertent error to our attention. The figure has now been replaced with a version created by the authors. This correction in no way affects the scientific claims made in this article.

